# Squamous Papilloma of the Soft Palate: A Case Report

**DOI:** 10.7759/cureus.37423

**Published:** 2023-04-11

**Authors:** Ghassan Darwish

**Affiliations:** 1 Oral and Maxillofacial Surgery, King Abdulaziz University, Faculty of Dentistry, Jeddah, SAU

**Keywords:** benign tumors, oral histopathology, oral soft tissue lesion, excisional tissue biopsy, squamous papilloma

## Abstract

Oral squamous papillomas (SPs) are benign masses commonly growing in the tongue, gingiva, uvula, lips, and palate. A case of an asymptomatic pedunculated squamous papilloma at the center of the soft palate is presented. Both surgical management and histopathologic analysis were conducted. The aim of this report is to stress the importance of early diagnosis and management of common benign oral lesions to prevent their transformation into malignancy.

## Introduction

Squamous papilloma (SP) is a common neoplasm of the oral cavity. It is a benign proliferation of stratified squamous epithelium that usually presents as a papillary or exophytic overgrowth in the oral cavity [1]. Its pathogenesis is mainly related to human papillomavirus (HPV) types 6 and 11 [2].

They occur in one in every 250 persons, most typically between the ages of 30 and 50, and may sometimes be seen in children below 10 years of age. Most investigations found no discernible gender predisposition or modest male predominance [3]. These neoplasms are slow-growing, nonaggressive lesions that are mainly present in the tongue, gingiva, vermilion of the lip, and palate [4]. They appear clinically as isolated solitary lesions in adults or multiple recurring in children. Recurrence of these lesions is rare and associated with a lower risk of malignant lesions [1,5]. However, early assessment and intervention are recommended, especially if these lesions appear during childhood [6]. Clinically, solitary oral squamous papillomas resemble verruca vulgaris, condyloma acuminatum, verruciform xanthoma, verrucous carcinoma, and papillary inflammatory hyperplasia [7].

Complete surgical excision of the base of the lesion, along with a small area of the surrounding tissue, is the treatment of choice to remove the lesion and prevent its recurrence [8].

Here, we present a case of squamous papilloma located at the center of the soft palate that had been ignored for more than 25 years. The lesion was managed by surgical excision and had a good prognosis based on histopathologic reports and follow-up appointments.

## Case presentation

**Figure 1:**
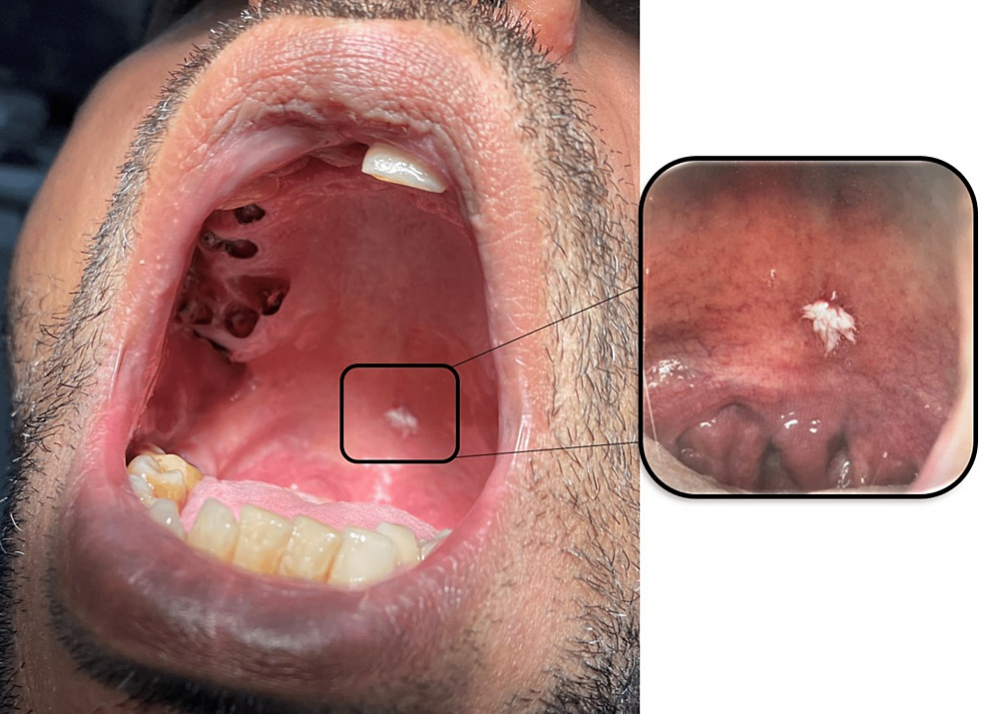
Intraoral image shows a white lesion at the center of the soft palate

**Figure 2:**
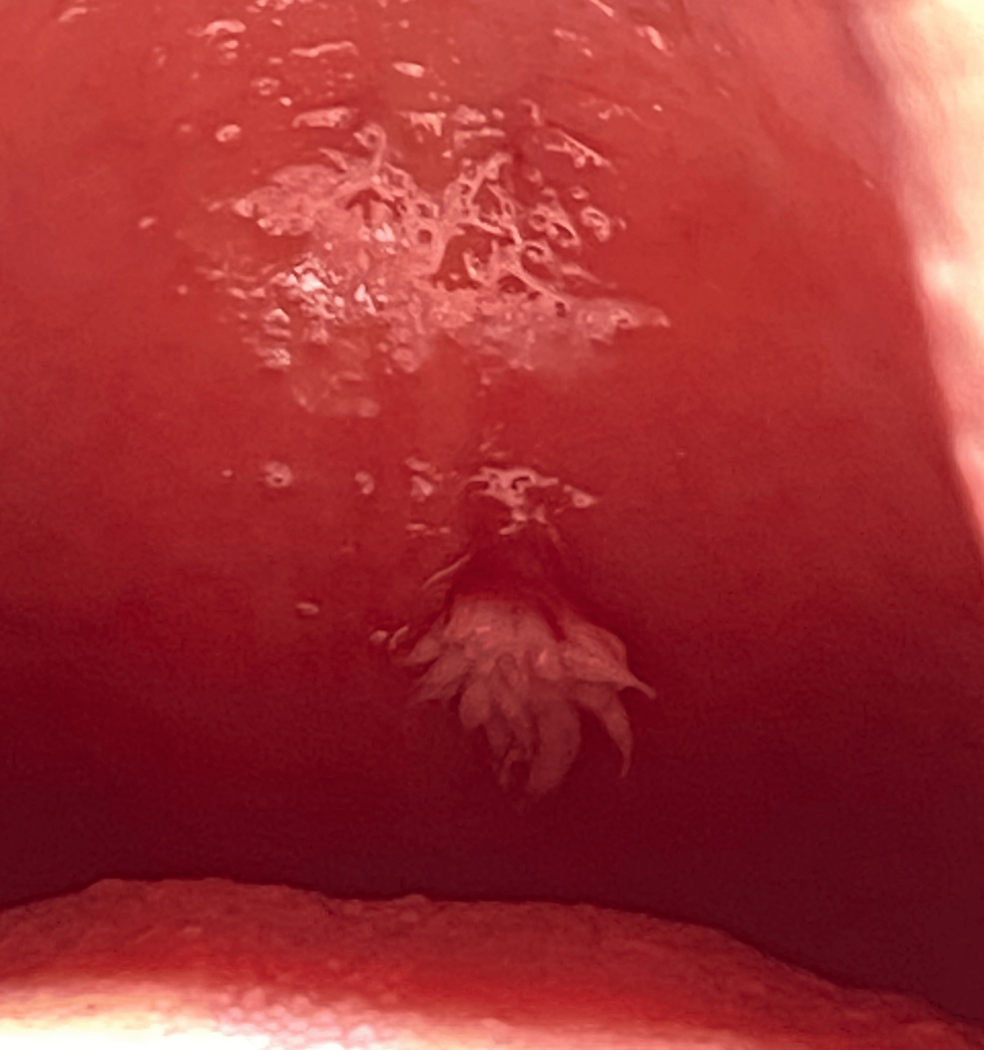
Intraoral image shows feather-like projections of the white lesion

A 36-year-old male with a medical history of anal fistula, gastroesophageal reflux, and left eye astigmatism, no known history of drug allergies, and a habit of smoking 25-30 cigarettes daily was referred to the oral and maxillofacial surgery clinic at King Abdulaziz University, School of Dentistry, to evaluate a soft tissue lesion associated with the soft palate. The patient noticed the lesion during childhood at 10 years of age and reported no history of pain, inflammation, or previous infection. Head and neck examination revealed no abnormalities, tenderness, or swelling. Intraoral examination revealed normal hard and soft tissues in the vestibule, buccal mucosa, floor of the mouth, and tongue. However, multiple non-restorable teeth were present. The intraoral image demonstrated a soft tissue lesion in the center of the soft palate (Figure 1). The lesion in the center of the soft palate was well-defined, warty, feather-like, white in color, and firm in consistency (Figure 2).

**Figure 3:**
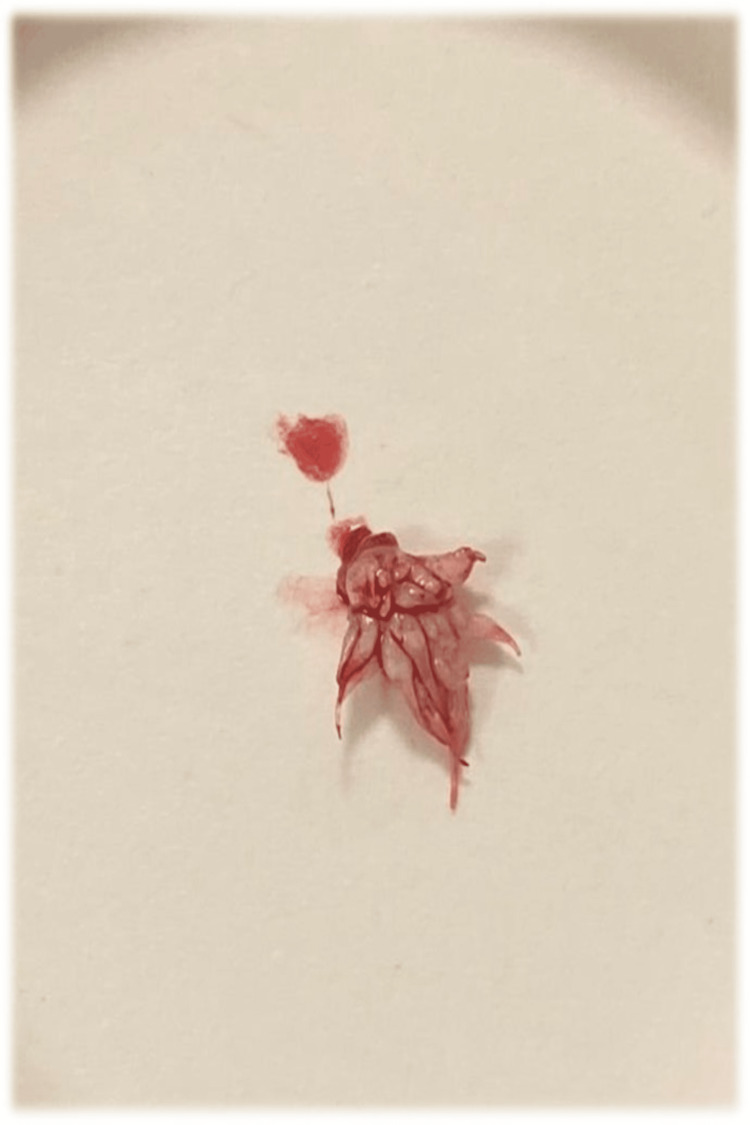
Image shows the surgically excised intraoral white lesion

The patient underwent a surgical excisional biopsy of the pedunculated soft palatal lesion under local anesthesia. One carpule of mepivacaine 2% with 1:100,000 epinephrine was used, and excision was performed using surgical blade number 15 from the base of the lesion. The lesion was then completely excised (Figure 3). The excision site was inspected and irrigated with normal saline, a 4-0 vicryl suture was placed, and hemostasis was achieved. The patient tolerated the procedure satisfactorily. The patient was recalled after one week and after three months for follow-up appointments. During follow-up, the patient denied pain or any discomfort. On clinical examination, no evidence of inflammation, discharge, infection, or recurrence of the lesion was observed.

**Figure 4:**
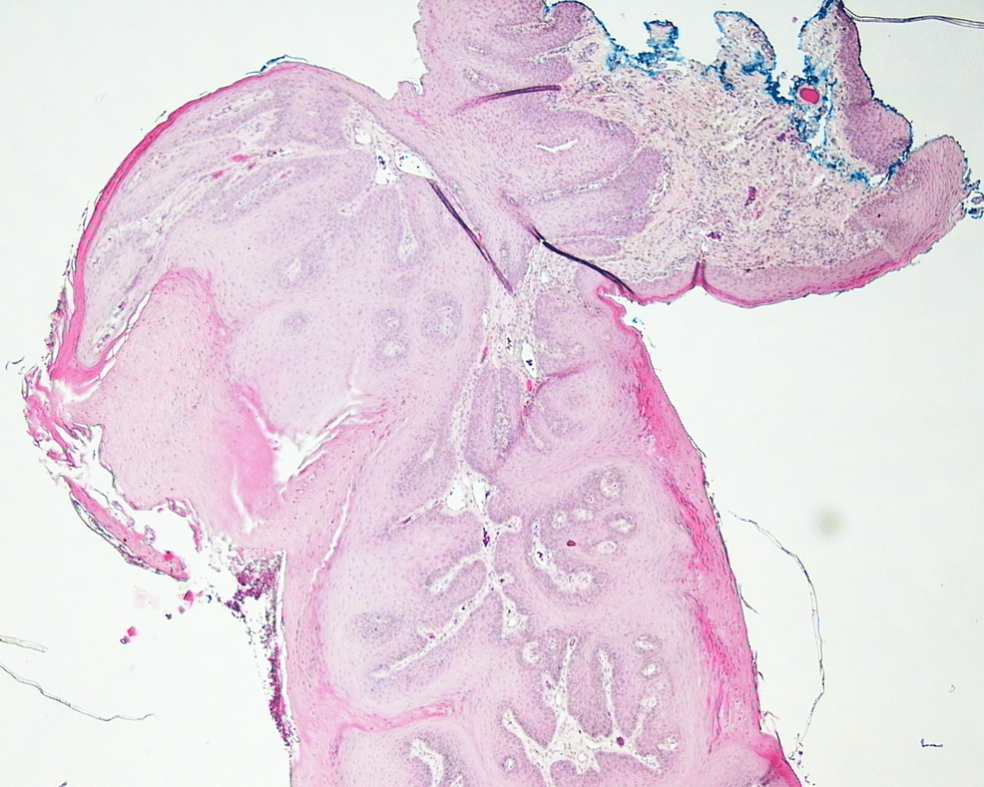
Histopathologic low-power image of the biopsy shows the lesion cores lined by parakeratinized stratified squamous epithelium

**Figure 5:**
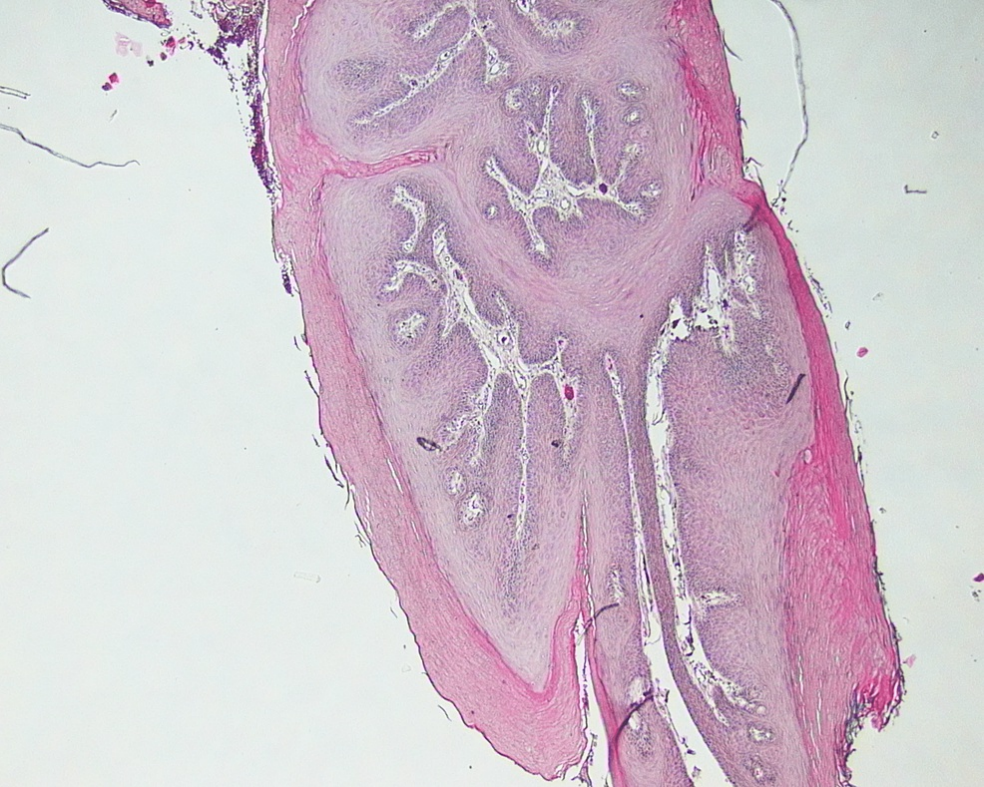
Histopathologic image shows hyperkeratosis with focal basal cell melanosis and melanin incontinence

Macroscopic histopathology examination revealed the presence of a specimen measuring approximately 0.9 cm × 0.5 cm × 0.2 cm, surfaced by parakeratinized stratified squamous epithelium (Figure 4). Epithelial hyperplasia forming finger-like projections with hyperkeratosis and vascularized fibrous connective tissue cores was reported, which confirmed the diagnosis of squamous papilloma. Focal basal cell melanosis and melanin incontinence were also observed (Figure 5).

## Discussion

According to the recent World Health Organization (WHO) classification, oral squamous papilloma is a benign hyperplastic exophytic localized overgrowth with a verrucous or cauliflower-like appearance with a sessile or pedunculated base [9]. The lesion measures approximately 1 cm in size, and the color may vary from pink to white depending on the level of keratinization and vascularity [10]. Oral squamous papilloma is characterized by slow growth and nonaggressive behavior and is commonly found in the tongue, palate, uvula, vermilion border of the lips, and gingiva. However, lesions present on the gingiva or those larger than 10 mm are at a higher risk of transforming into malignancy [10].

In our case, the patient presented with a soft lesion in the center of the soft palate. The clinical appearance of the lesion on the soft palate was well-defined, warty, feather-like, white in color, and firm in consistency. These findings match the clinical appearance of squamous papillomas reported in the literature [5]. Although the patient noticed this lesion at 10 years of age, he reported it at 36 years of age. Squamous papillomas are commonly reported in the patient age group of 30-50 years. They may also be seen in children aged <10 years and contribute to 8% of all oral tumors in children [11]. However, in a study by Frigerio et al. [7], a large variability in age was observed as papillomas were diagnosed in a young five-year-old patient and the oldest 92-year-old patient. The mean age of the patients was reported to be 48.5 years. The gender predilection for this condition remains controversial, but research suggests that this lesion may be more common in men than in women [12].

Several factors, such as tobacco use, alcohol consumption, hormonal changes, and immunosuppression, are associated with the development of oral squamous papilloma. Moreover, certain strains of human papillomavirus, particularly HPV-6 and HPV-11, have been implicated in the development of oral squamous papilloma [1]. In our case, the patient was a chronic smoker with a habit of smoking 25-30 cigarettes daily, which could be a contributing factor to oral squamous papilloma. However, the patient first noticed the lesion at the age of 10 years. Therefore, a direct relationship between tobacco use and oral squamous development could not be established in our study. These lesions are generally non-transmissible; however, literature associates these lesions with highly contagious HPV-6 and HPV-11 strains of human papillomavirus (HPV). The exact cause of the development of squamous papilloma remains controversial. For instance, Harries et al. suggested that this condition could be due to a response to tissue injury rather than a tumor. Subsequently, this theory was rejected, and a definite association was established between HPV and the development of squamous papilloma. However, some authors consider the association between squamous papilloma and HPV as a coincidental finding [13,14].

Based on the clinical features of the lesion, a differential diagnosis of papillary hyperplasia, verruciform xanthoma, condyloma acuminatum, and focal epithelial hyperplasia was suggested. Although papillary hyperplasia resembles squamous papilloma, no evidence of a soft tissue reaction to chronic irritation or injury was evident in our study, which is a common reason for the development of hyperplasia. Verruciform xanthomas are commonly found in the gingiva and alveolar ridges. Condylomas are large and have a broader base than papillomas with a pink-red color instead of white. Additionally, focal epithelial hyperplasia resembles multiple squamous papillomas instead of solitary lesions, as observed in our study [14]. The final diagnosis of the lesion in our study was confirmed by histopathologic examination, which showed a hyperplastic squamous epithelium with a fibrovascular connective tissue core.

The presence of epithelial hyperplasia forming finger-like projections gives the lesion a cauliflower-like appearance. These lesions are treated using routine surgical excision or laser ablation [8]. Other treatment modalities include electrocautery, cryosurgery, and intralesional interferon injections [15]. In this patient, a surgical excisional biopsy was performed without any complications. Although the recurrence rate is very low, if it happens, it will be before 15 months of follow-up [2]. Therefore, follow-up appointments should be arranged at three-month intervals for up to a year and a half to detect any early lesion recurrence.

Asymptomatic lesions are a common finding when intraoral evaluation is performed, but in some cases, the lesion shows a malignant resemblance or transformation to oral squamous cell carcinoma [1]. Therefore, they should be detected early and carefully analyzed histopathologically [10]. The present study’s age of occurrence, gender predilection, and lesion characteristics both clinically and histologically agree with the literature.

## Conclusions

Squamous papillomas were classified as benign. Although papillomas are usually nonaggressive and asymptomatic, symptomatic squamous papillomas have been reported. Because the lesions may resemble or transform into oral squamous cell carcinoma, a much more aggressive tumor, rigorous histopathologic assessment of these lesions is necessary. Therefore, prompt diagnosis and surgical removal of the lesion will provide comfort to the patient and avoid subsequent complications.
